# More than Just a Narrative: Measuring People’s Experience of Care Coordination to Improve Quality and Outcomes

**DOI:** 10.5334/ijic.3077

**Published:** 2017-03-31

**Authors:** Nick Goodwin

**Affiliations:** 1 International Foundation for Integrated Care, Wolfson College, Oxford, UK

One of the key insights from the experience of those implementing integrated care is the ability to develop a clear understanding of what integrated care means for all those involved. A common strategy has been to develop a compelling narrative that helps to explain the rationale that inspires care providers and professionals to think and act differently. In England, for example, ‘the narrative’ developed by National Voices, a non-profit organisation representing the views of patients and patient groups, articulated a national vision for person-centred coordinated care that was adopted at the highest political level as the underpinning definition for the country’s integrated care strategy [1].

However, the experience in England and other countries is that there appears to be a considerable difference between adopting such a narrative and seeing this translate into policy or practice. For example, a recent report from the Project INTEGRATE research programme found that the ‘person-centred’ perspective was almost always absent when it came to a content analysis of the real motivations behind integration policies across Europe [2]. These policies were mostly dominated by the need to make cost efficiencies.

In my last IJIC editorial the point was made that many efforts to promote integrated care may be becoming self-serving (e.g. to address a policy imperative) rather than being the mechanism through which to improve people’s care and outcomes [3]. In England’s most recent set of reforms (known as *Sustainability and Transformation Plans*) there has been a new drive for significantly more ambitious, population-based, initiatives that introduce new models of integrated health and social care with the purpose of improving health and wellbeing as well as the efficiency of services [4]. However, the evidence suggests the operational reality has been to focus on the immediate problems related to workforce, productivity, managing acute demand and closing gaps in available finances [5].

The English experience is mirrored across many health and care systems that are under severe pressure to manage increasing costs of care within tight financial constraints. Yet in the process of tackling such challenges the defining ‘narrative’ for integrated care becomes either skewed or lost. The users’ voice has been effectively drowned out and so the potential to engage and empower people and communities is increasingly overlooked.

The threat this poses to the success, or otherwise, of integrated care programs cannot be overstated. If the focus of integration becomes placed overmuch on organisational solutions, as opposed to new ways of working for the benefit of service users, then the evidence tends to demonstrate limited results. For example, the National Audit Office’s recent report demonstrates that despite so much effort to promote the integration of the health and social care sector in England the benefits have fallen far short of expectations. Financial pressures have meant there is little hope that the sector can deliver on their commitments to integrate services by 2020 [6].

The logical observation to be drawn is that the ability to promote the person-centred narrative may always be crowded out by economics. However, I would suggest that one of the contributing factors behind this observation is that decision-makers find it difficult to understand the financial value of measuring people’s care experiences to inform and drive forward more effective care design processes.

Measuring people’s experiences is important not only to guide service improvement, but also because people’s experiences of care has shown to be linked to improved clinical outcomes and reduced costs. For example, a systematic review of 55 studies in primary care and hospitals found consistent positive associations between patient experience, patient safety and clinical effectiveness for a wide range of disease areas, settings, outcome measures and study designs [7]. In theory, measuring care experiences should encourage better decision-making and lead to more effective service delivery.

However, measuring people’s experiences of integrated care is a problem because care is provided from a more complex mix of care professionals and providers. Few survey tools have sought to capture the experiences of people with complex chronic conditions to assess how well care is being co-ordinated around their needs. In this edition of IJIC, Crump *et al* report on the promising development of such a survey tool that gathers user reported measures of care co-ordination with the intention to support quality improvements in practice [8]. Though the tool performed well during piloting, the authors argue it would be used best to support discussions related to quality improvement but not for performance assessment.

If people’s experiences are to really underpin the narrative that justifies the rationale for integration then more needs to be done to develop surveys and tools of the kind created by Crump *et al* [8]. The next step on from that will be to ascertain the connection between survey results, costs and outcomes. It is then more likely for measures of care experiences to be embedded in performance frameworks since they offer a contribution to supporting the ‘bottom line’ financial costs that predominate decision-making. If the maxim ‘you cannot improve what you cannot measure’ is to be believed then this helps to predict why a person-centred narrative without the means to assess its value is often likely to remain political rhetoric rather than operational reality.
